# Cumulative Household Food Insecurity Is Associated with Adverse Eating Behaviors Among Youth Exposed to Gestational Diabetes in Utero

**DOI:** 10.3390/nu18172827

**Published:** 2026-08-28

**Authors:** Keally Haushalter, Jaimie N. Davis, Myles S. Faith, Yeyi Zhu, Erin A. Hudson, Alexis S. King, Erica P. Gunderson

**Affiliations:** 1 Department of Nutritional Sciences, The University of Texas at Austin, Austin, TX 78723, USA; 2 School and Educational Psychology, The University of Buffalo, Buffalo, NY 14214, USA; 3 Kaiser Permanente Northern California Division of Research, Pleasanton, CA 94588, USA; 4 Department of Health Systems Science, Kaiser Permanente Bernard J. Tyson School of Medicine, Pasadena, CA 91101, USA

**Keywords:** food insecurity, eating behaviors, gestational diabetes, food approach, breastfeeding duration, breastfeeding exclusivity

## Abstract

Background/Objectives: Food insecurity has been associated with adverse eating patterns, which increase the risk for overweight and obesity. However, most studies measure food insecurity and eating behaviors cross-sectionally, not assessing whether experiencing food insecurity over time influences eating behaviors. Therefore, this study assessed the relationship between cumulative food insecurity and food approach and food avoidance eating behaviors and evaluated the effect of breastfeeding intensity and duration on this relationship. Methods: The Study of Women, Infant Feeding and Type 2 Diabetes after Gestational Diabetes Pregnancy (SWIFT) is a prospective longitudinal cohort of 1033 mothers diagnosed with gestational diabetes and their youth. Breastfeeding intensity and duration were assessed at research visits and through mailed monthly surveys from birth to one year postpartum. The SWIFT study in Youth (SWIFT-Y) was a cohort of 701 SWIFT offspring that consented to participate in a research visit 11–16 years postpartum. Youth’s BMI was measured at 11–16 years old through a research visit or their electronic health record. At each of the four research visits (6–9 weeks, one year, two years, and 11–16 years postpartum), participants were categorized as being food secure (0) or food insecure (1). Scores across the four visits were summed (range 0–4), reflecting the number of assessments one was classified as food insecure. The Child Eating Behavior Questionnaire was used to measure the prevalence of eight eating behaviors in the youth, with higher scores indicating that the behaviors were more apparent. According to the measure, the behaviors were categorized into two groups: food approach and food avoidance. Food approach consists of youth’s food responsiveness, emotional overeating, enjoyment of food, and desire to drink, and has been positively associated with a child’s BMI. Food avoidance includes the youth’s satiety responsiveness, slowness in eating, emotional undereating, and food fussiness, and is negatively associated with BMI. Linear regressions assessed whether cumulative food insecurity was associated with eating patterns, and exploratory analysis analyzed whether this relationship differed by breastfeeding duration (<six months, ≥six months) and intensity (exclusive, not exclusive), controlling for the child’s BMI and sociodemographic characteristics. Results: In this complete-case secondary analysis (*n* = 321), each additional experience of food insecurity was associated with a higher food approach score (B = 0.30, 95% CI [0.04, 0.57], *p* = 0.03), but not food avoidance (B = −0.03, 95% CI [−0.21, 0.15], *p* = 0.74). These results varied by breastfeeding duration (B = −0.74, *p* = 0.001), as the relationship remained significant among those who were breastfed for <six months (B = 0.73, *p* < 0.001) but was attenuated among those who were breastfed for ≥six months (B = −0.26, *p* = 0.18). Conclusions: Experiencing food insecurity more during childhood is associated with adverse eating patterns at 11–16 years old. However, breastfeeding for ≥six months influenced this relationship, suggesting that early-life feeding may impact the effect of food insecurity on one’s eating behaviors later in life.

## 1. Introduction

In 2024, food insecurity affected 18.3 million (13.7%) households in the United States (US) [1]. The condition, defined by the US Department of Agriculture (USDA) as “a household-level economic and social condition of limited or uncertain access to adequate food”, has been associated with multiple adverse diet-related health outcomes [2]. In adults, food insecurity is related to a higher risk of poor health, diabetes, hypertension, overweight, and obesity, and if experienced during pregnancy, a higher risk of gestational diabetes [3,4]. Children with food insecurity have a higher risk of multiple diet-related conditions, including diabetes, overweight, and obesity [5,6].

One possible explanation for the relationship between food insecurity and diet-related chronic conditions among children is eating patterns. A systematic review found that children who experienced food insecurity were more likely to eat in the absence of hunger, binge eat, and have loss-of-control when eating [7]. This is concerning as these eating behaviors are associated with a higher risk for adverse health conditions, including overweight and obesity [8,9,10]. Children’s eating patterns are commonly measured with the Child Eating Behavior Questionnaire (CEBQ), a validated questionnaire in which parents report their children’s eating behaviors across eight domains. According to the measure’s guidelines, the eight domains are grouped into two composite scores, “food approach” and “food avoidance” [11].

Food approach consists of the four domains: food responsiveness, emotional overeating, enjoyment of food, and desire to drink, and has been positively associated with a child’s BMI and binge-eating behaviors [12,13]. Food responsiveness measures the child’s desire for food, regardless of whether food is present [11]. Emotional overeating assesses whether a child tends to eat more during a heightened positive or negative emotional state [11]. Enjoyment of food captures how much the child enjoys and looks forward to eating [11]. Desire to drink describes whether the child likes to have a drink at mealtimes and throughout the day [11].

Food avoidance consists of four domains: satiety responsiveness, slowness in eating, emotional undereating, and food fussiness, and has been negatively associated with a child’s BMI [11,13]. Satiety responsiveness is how much a child eats [11]. Slowness in eating describes how fast a child eats [11]. Emotional undereating assesses whether a child consumes less depending on their mood, and food fussiness is how selective or accepting a child is of new foods [11].

Research analyzing how food insecurity impacts children’s eating behaviors has yielded mixed results [14,15,16]. For example, a cross-sectional study of 66 primarily low-income Hispanic youth found that children in food-insecure households had higher food responsiveness and enjoyment of food scores and lower satiety responsiveness scores [15]. On the other hand, baseline data from a study of 822 2–18-year-olds with obesity found that food insecurity was positively associated only with the desire to drink [14]. Further, a cross-sectional analysis of 2404 five-year-olds found that those with low socioeconomic status had higher food responsiveness, desire to drink, and emotional overeating scores [16]. However, more research on how food insecurity impacts overall eating behaviors, as exemplified through the food approach and food avoidance scores, is needed to provide a more holistic understanding of this relationship.

In addition, most of the studies that analyze the impact of food insecurity and eating behaviors are cross-sectional [7]. This is concerning as food insecurity is a dynamic condition, and most people who experience food insecurity also experience periods of food security [17]. Therefore, measuring and characterizing one’s food insecurity status at one time fails to capture their dynamic experience with food insecurity [7]. Second, not all the studies control for the child’s BMI [7]. Without controlling for the child’s BMI, the relationship between food insecurity and eating behaviors cannot be disaggregated, as their adverse eating patterns may reflect their BMI, not eating patterns related to experiencing food insecurity per se.

While food-insecure children have a higher risk for developing adverse health conditions, those exposed to gestational diabetes in utero, a condition in which pregnant women develop elevated blood glucose during pregnancy, also have a higher risk for adverse health outcomes [18,19]. Gestational diabetes, which impacts approximately one in ten pregnancies in the US, exposes the fetus to high blood glucose, creating an adverse intrauterine environment that can potentially alter fetal programming [20,21]. As a result, children exposed to gestational diabetes in utero have a higher risk for developing health conditions, such as obesity and insulin resistance, later in life [18,19]. Our prior research has found that exclusive breastfeeding and breastfeeding for longer may reduce the offspring’s risk for developing the adverse outcomes associated with exposure to gestational diabetes [22,23]. However, it is unclear if the protective nature of breastfeeding persists among youth exposed to gestational diabetes in utero who also experience food insecurity during childhood.

Therefore, the objective of this study is two-fold. The first objective was to analyze whether cumulative food insecurity from birth to 11–16 years old was associated with food avoidance and food approach behaviors at 11–16 years old, in unadjusted and adjusted analysis that controlled for BMI, among a cohort of offspring exposed to gestational diabetes in utero. As an exploratory aim, given that cumulative food insecurity was significantly associated with food approach scores, the second objective examined if this relationship differed by breastfeeding intensity and breastfeeding duration. Finally, the association between current food insecurity status and food approach scores was examined as a sensitivity analysis to assess if current food insecurity, rather than cumulative food insecurity, could explain this relationship.

We hypothesized that experiencing food insecurity more often would be associated with higher food approach scores and lower food avoidance scores, but this relationship would be attenuated by those who breastfed exclusively for six months or longer. Finally, we hypothesized that this relationship would not remain when only assessing current food insecurity status, not cumulative food insecurity.

## 2. Materials and Methods

### 2.1. Study Overview

Data from the Study of Women, Infant Feeding and Type 2 Diabetes after Gestational Diabetes Pregnancy (SWIFT) and the SWIFT Study in Youth (SWIFT-Y) were used for this secondary analysis, and detailed descriptions of the studies are published elsewhere [24,25]. Briefly, SWIFT is a prospective cohort of 1033 mother-child dyads diagnosed with gestational diabetes using the Carpenter and Coustan criteria [26], who delivered a healthy, live singleton at ≥35 weeks of gestation at a Kaiser Permanente Northern California hospital (2008–2011), aged 20–45 years old at delivery and met study eligibility criteria [24]. At study baseline, 1033 mothers consented to participate in three in-person research examinations (at 6–9 weeks, one year, and two years postpartum), which included metabolic, anthropometric, medical history, reproductive, sociodemographic, psychosocial, and lifestyle behavior measures, and, during the first year postpartum, participants completed monthly mailed surveys regarding infant feeding and health [24]. Of the 1033 participants, 1031 mother-infant dyads completed first-year breastfeeding intensity and duration surveys. In 2022–2025, SWIFT women were recontacted to participate in a fourth research visit, which included similar metabolic, anthropometric, and other measures, and to enroll youth aged 11–16 years old who consented to the SWIFT-Y in-person research visit to evaluate behaviors and health outcomes. Of the 701 mother-child dyads enrolled in SWIFT-Y, 552 completed the CEBQ, and 364 mothers previously completed all surveys needed to calculate food insecurity across all four SWIFT research visits from 2008–2025. Of the SWIFT-Y dyads who completed all food insecurity surveys and the CEBQ, 321 youth also had their height and weight measured six months before or after they assented to participate in SWIFT-Y. Most youth had their height and weight measured at the in-person SWIFT-Y research visit (*n* = 275), but if they did not complete their SWIFT-Y in-person research visit, their clinical height and weight from the Kaiser Permanente Northern California electronic health records were used (*n* = 46). The SWIFT and SWIFT-Y studies were approved by the Institutional Review Board at Kaiser Permanente Northern California and registered as a clinical trial (NCT019967030). Measures relevant to this secondary analysis are described below.

### 2.2. Food Insecurity

Food insecurity was assessed using the USDA Food Sufficiency Questionnaire/Screener, which combines the participant’s answer to the screener with their income-to-poverty ratio to categorize them as food secure or food insecure [27]. This survey has been used in multiple national studies, including the Nationwide Food Consumption Survey and the Food Security Supplement to the Census Bureau’s Current Population Survey, and has been previously correlated with other measures of food insecurity [28,29]. Mothers completed the food security screener, household income, and household size at all four research visits (6–9 weeks, one year, two years, and 11–16 years postpartum). Only those who completed these measures at all four of the visits were included in this analysis.

The USDA Food Sufficiency Question/Screener which asks the mothers “Within the past 12 months, which one of these statements best describes the food eaten by your family?” with answer options of: “We have enough food to eat and the kinds of food we want”, “We have enough food to eat, but NOT always the kinds of food we want to eat”, “Sometimes we don’t have enough food to eat”, “Often we don’t have enough food to eat,” and “Prefer not to answer” [24,25,27]. Mothers reported their household income through the question “What was your total combined income for ALL family members in your household for the past 12 months from all sources, before taxes?” and selected which income bracket they fell into (ranging from <5000 to >150,000) [24,25]. Mothers also reported how many individuals lived in their homes by writing a numeric answer to the question, “How many persons currently live in your home, including yourself?” [24,25].

The participant’s poverty-to-income ratio was calculated using the household size, income, and the respective poverty guideline from the year the survey was completed, by dividing their income by the poverty level for their respective household size. Following the USDA guidelines, at each visit, participants were then classified as food secure if they answered, “We have enough food to eat and the kinds of food we want,” and had an income-to-poverty ratio greater than two. If participants answered, “We have enough food to eat, but NOT always the kinds of food we want to eat”, “Sometimes we don’t have enough food to eat”, or “Often we don’t have enough food to eat”, they were classified as food insecure regardless of income-to-poverty ratio [27]. Finally, in line with the guidelines, if participants selected “We have enough food to eat and the kinds of food we want,” but had an income-to-poverty ratio equal to or less than two, they were classified as food insecure [27].

For each visit, participants were assigned a one if they were food insecure and a zero if they were food secure. To assess cumulative food insecurity, the scores were summed across all four visits for each participant, ranging from zero to four. A zero indicated that the participant was never food insecure, while a four indicated that the participant was food insecure at all four visits (i.e., a higher score indicates experiencing food insecurity more).

### 2.3. Child Eating Behaviors

Child eating behaviors were reported by mothers using the CEBQ at SWIFT-Y research visit 1 [11]. The CEBQ consists of 35 questions in which parents indicate on a five-point Likert scale if their child “never”, “rarely”, “sometimes”, “often”, or “always” does the given behavior [11]. The answers are converted to numeric values, in which doing the behavior more often is scored higher (never = 1, rarely = 2, sometimes = 3, often = 4, always = 5) [11]. Eight domains (food responsiveness, emotional overeating, enjoyment of food, desire to drink, satiety responsiveness, slowness in eating, emotional undereating, and food fussiness) are derived from the questions, and a higher mean domain score indicates the child doing the domain behavior more frequently [11]. In line with previous research [13], mean scores of food responsiveness, emotional overeating, enjoyment of food, and desire to drink were added together to create the composite mean score for “food approach” (range 4–20), with higher scores reflecting doing the behaviors more frequently. Satiety responsiveness, slowness in eating, emotional undereating, and food fussiness were summed to create the composite mean score for “food avoidance” (range 4–20), with higher scores indicating that the child did the behaviors more frequently. Since its original development and validation among parents of youth aged 6–7 years old, the measure has been validated in parents of youth aged 6–16 years old, demonstrating that it has internal consistency and reliability in an older sample [30]. In addition, among the older sample, the measure displayed similar relationships with BMI (positive associations among all food approach domains and negative associations among all the food avoidance domains, except for emotional undereating) [30]. In the analytic sample, the food approach domain had a Cronbach alpha value of 0.73 and the food avoidance domain had a Cronbach alpha value of 0.53 [31].

### 2.4. Youth’s BMI

Youths’ height and weight measurements were obtained from two sources. If the youth completed their SWIFT-Y research visit (2022–2025, 11–16 years old), their height and weight were measured by research staff (*n* = 275). Youths’ height was measured to the nearest 0.5 cm using a stadiometer, and their weight was measured to the nearest 0.1 kg using a calibrated research digital scale [25]. If the child did not attend their research visit, their electronic health record was used to obtain their height and weight, which were assessed at a Kaiser Permanente Northern California routine well-child outpatient visit using standard clinical practices (*n* = 46). Measurements within a one-year range (six months before or after) of when the youth assented to participate in the study were used, and if the child had multiple visits that occurred within that range, the visit closest to the assent date was selected. The youth’s BMI percentile was determined using the Centers for Disease Control and Prevention age- and sex-specific growth charts [32].

### 2.5. Breastfeeding Intensity and Duration

Breastfeeding intensity and duration were assessed through detailed infant feeding surveys that mothers completed at the first three in-person SWIFT research visits (6–9 weeks, one year, and two years postpartum), which were administered by research staff, and through returned surveys that were mailed to them monthly for the first year [24,25]. In each survey, mothers reported the average number of breastmilk feeds, formula feeds, and total feeds in the past week [24,25]. Mothers also reported if they ever breastfed, if they were currently breastfeeding, and if they were not currently breastfeeding, when and why they discontinued breastfeeding [24,25].

To quantify lactation intensity and duration, as described in previous SWIFT publications, participants reported the average number of breastmilk feeds during the past seven days and were assigned a score between zero and one each month based on the percentage of feeds that month that were breastmilk [24,25,33]. A score of zero indicated that none of the feedings were breastmilk, and a score of one indicated that 100% of the feedings were breastmilk. The lactation duration and intensity ratio summary score was created by summing the monthly scores from birth to 12 months to provide a single continuous measure of breastfeeding intensity and duration across the first year [24,25,33]. The score ranged from zero to 12, with a higher score indicating more intense and longer breastfeeding [24,25,33].

Breastfeeding intensity and duration were also dichotomized according to the World Health Organization recommendations and in line with other SWIFT analyses [22,23,24,25,34,35,36,37]. Participants were classified into the “exclusive breastfeeding” group if 100% of their feeds were breastmilk from birth to six months and they were not fed any water, other liquids, or complementary foods. Participants were classified as “not exclusive breastfeeding” if <100% of their feeds were breastmilk from birth to six months, including if they were fed water, other liquids, or complementary foods. Breastfeeding duration was dichotomized as <six months and ≥six months.

### 2.6. Covariates

Potential covariates were considered based on a priori hypotheses and previously published findings [7,13,14,15,22,23,35]. At the 6–9 weeks postpartum research visit, mothers reported their educational attainment (less than college, college degree, or more) and nativity (born in the US, born outside the US/Unknown), and child’s race and ethnicity (White, Asian, Hispanic, Black/Multiracial/Other) through surveys. The mother’s Kaiser Permanente Northern California electronic health record was used to obtain their pre-pregnancy height and weight to calculate pre-pregnancy BMI, gestational age at gestational diabetes diagnosis, pregnancy course and perinatal outcomes, and child’s sex and birth date. At the 6–9 weeks, one-year, and two-year in-person research visits, mothers reported whether they participated in The Special Supplemental Nutrition Program for Women, Infants, and Children (WIC) through the question, “Do you receive benefits from the WIC program?” with answer options of “no”, “yes”, and “no answer”. If participants selected “yes” at any of the three visits, they were categorized as a “WIC Recipient.”

### 2.7. Statistical Analysis

The analytic sample consisted of youth with complete data on all study measures, including food insecurity from mothers’ research visits 1–4, CEBQ at youth’s research visit 1, and height and weight measured either at the youth’s SWIFT-Y research visit 1 (*n* = 275) or at a routine well-child visit within one year of assenting to SWIFT-Y (*n* = 46). Inferential statistics (*t*-test for normally distributed continuous variables, Wilcoxon rank sum for non-normally distributed continuous variables, and chi-square tests for categorical variables) were used to assess significant differences between those in the SWIFT participants not included in the analytic sample (*n* = 712) and those included in the analytic sample (*n* = 321).

To test our first study objective, linear regressions were used to assess how cumulative food insecurity was associated with the child’s food approach score, first controlling for nothing (unadjusted model), and then controlling for the child’s BMI percentile, race and ethnicity, age, sex, lactation intensity ratio, mother’s pre-pregnancy BMI, mother’s education attainment, WIC status, and mother’s nativity (adjusted model). Similarly, we ran parallel unadjusted and adjusted models to assess the relationship between cumulative food insecurity and food avoidance score. Results were considered significant at *p* < 0.05.

To test our second exploratory aim, a multivariable linear regression assessed the interaction between cumulative food insecurity and the lactation intensity ratio on food approach and food avoidance, separately. As the interaction between cumulative food insecurity and lactation intensity on food approach was significant, lactation intensity was further categorized by breastfeeding exclusivity (exclusive vs. not exclusive) and duration (<six months vs. ≥six months). The interaction between cumulative food insecurity and breastfeeding exclusivity on food approach was assessed with a multivariable linear regression, adjusting for the same covariates used previously. The same method was used to assess the interaction between cumulative food insecurity and breastfeeding duration on food approach scores. Since the interaction between cumulative food insecurity and breastfeeding duration on food approach scores was significant, a multivariable regression was used to assess the relationship between cumulative food insecurity and food approach scores, stratified by breastfeeding duration groups (<six months vs. ≥six months). To account for multiple testing across the five exploratory analyses, a Bonferroni correction was applied, and significance was considered at *p* < 0.01.

As a sensitivity analysis, unadjusted and adjusted multivariable linear regressions assessed the relationship between current food insecurity status (assessed at the 11–16-year-old research visit) and food approach scores, adjusting for the same covariates, to ensure the relationship between food insecurity and food approach scores reflected cumulative food insecurity, not current food insecurity status.

Assumptions of linear regression were evaluated for the primary, interaction, and exploratory analyses. Residual-versus-fitted plots were used to assess nonlinearity, Q-Q plots assessed the normality of residuals, scale-location plots assessed heteroskedasticity, and residual-versus-leverage plots examined influential observations. Using ANOVA, linearity was assessed by comparing the model fits of the fully adjusted model with food insecurity as a continuous variable versus a categorical variable. The categorical specification did not significantly improve model fit (food approach: *p* = 0.17, food avoidance: *p* = 0.39), so food insecurity was kept as a continuous variable. All analyses were conducted in RStudio (Version 2023.09 “Desert Sunflower” Release (b51c81cc, 25 September 2023) for Windows, Vienna, Austria).

## 3. Results

### 3.1. Characteristics of the Analytic Sample

**Figure 1:**
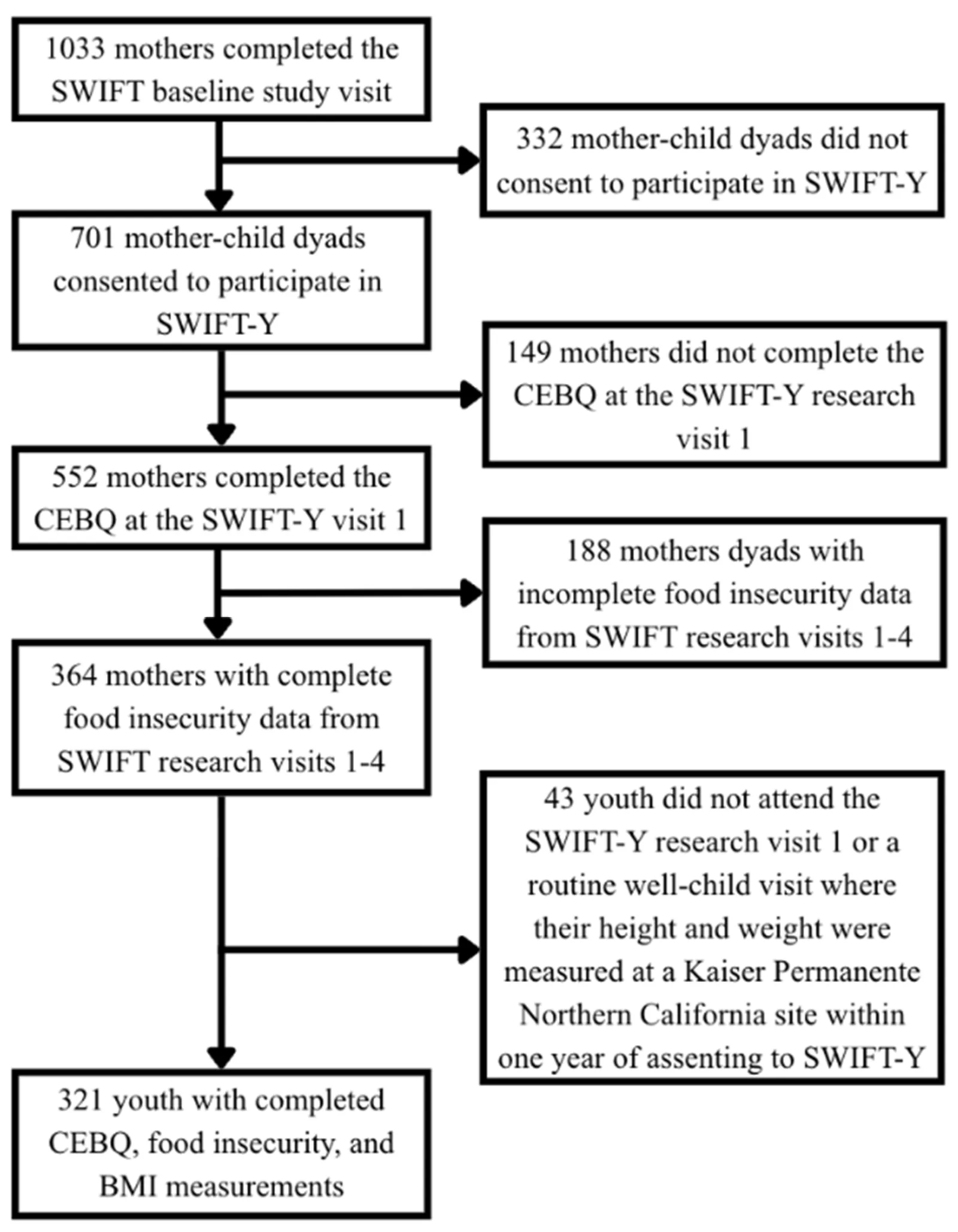
Flow diagram of the analytic sample.

The analytic sample comprised 321 youth with complete data on food insecurity, eating behavior, and BMI, presented in Figure 1. The significant differences in sociodemographic characteristics between the SWIFT participants included in the analytic sample and those excluded are presented in Table 1. Compared to the SWIFT participants excluded from the analytic sample, those in the analytic sample had a higher average lactation intensity ratio score (6.8 vs. 5.3, *p* < 0.001), a higher percentage were breastfed for six months or longer (66.0% vs. 53.7%, *p* < 0.001), and a higher percentage of mothers with a college degree or more (56.1% vs. 45.5%, *p* = 0.002). No other sociodemographic variables differed significantly between those included in the analytic sample and those who were not.

On average (±standard deviation), those in the analytic sample were 13.5 (±1.0) years old, 44.6% were female, 35.5% were Asian, and 34.3% were Hispanic. Mothers had an average pre-pregnancy BMI of 29.2 kg/m^2^ (±7.0), and one-third (31.5%) received WIC at some point during their pregnancy or the first two years postpartum. Youth were food insecure for an average of 0.81 (±1.3) times, with 202 (62.9%) never experiencing food insecurity, 45 (14.0%) experiencing food insecurity once, 30 (9.3%) experiencing food insecurity twice, 20 (6.2%) experiencing food insecurity three times, and 24 (7.5%) experiencing food insecurity all four times. Youth had an average BMI percentile of 69.0 (±29.1), an average food approach score of 10.7 (±2.4), and an average food avoidance score of 8.0 (±1.6). Approximately 66% of the participants were breastfed for six months or longer (*n* = 212), and 25% (*n* = 79) were exclusively breastfed.

**Figure 2:**
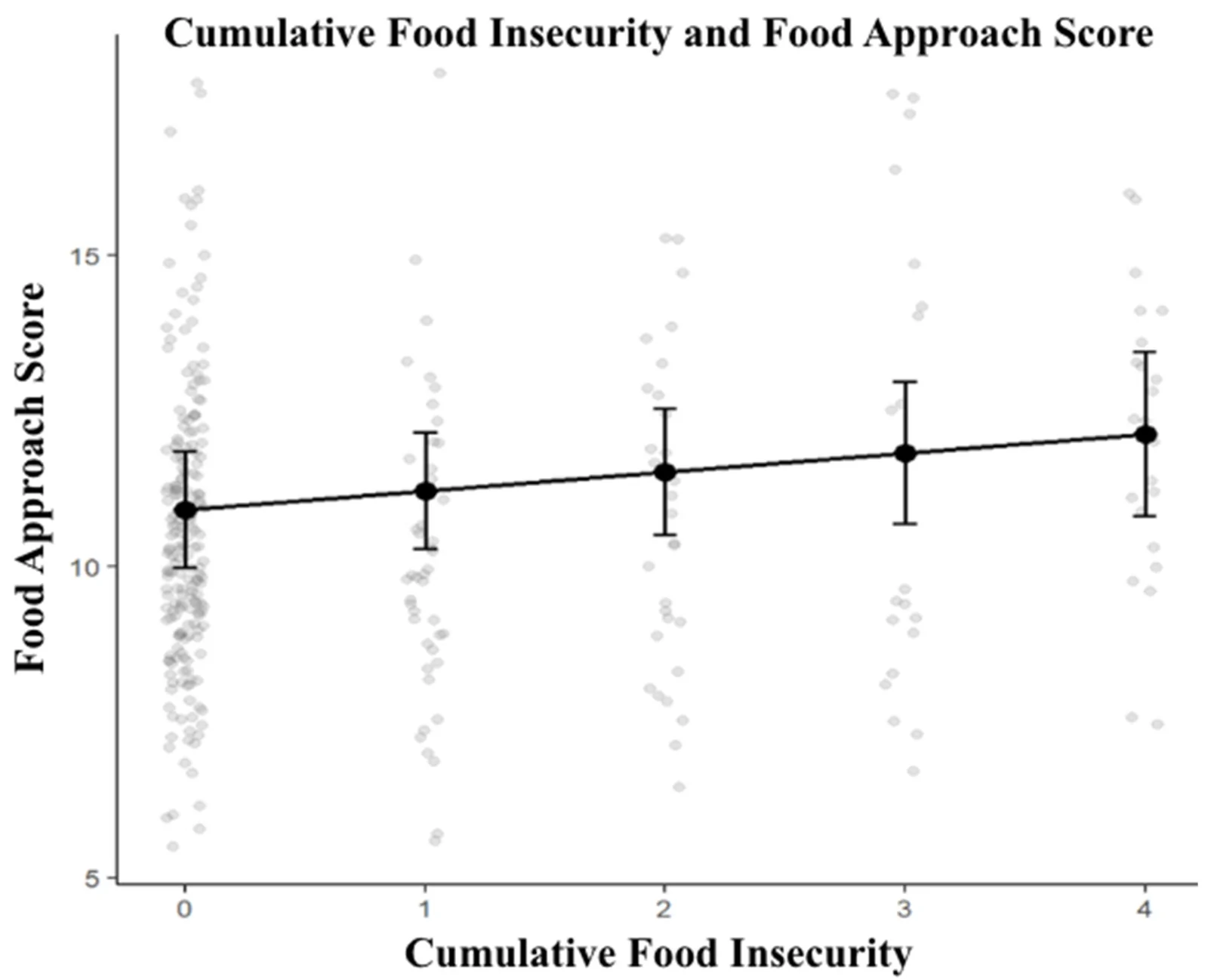
Cumulative food insecurity and food approach score. The grey dots represent the observed data and the solid line represents the average marginal predicted mean and the 95% confidence interval of food approach scores, adjusting for the mean values (±95% confidence interval) for continuous covariates (BMI percentile, age, lactation intensity ratio, mother’s pre-pregnancy BMI) and reference group for categorical variables (sex = female, race and ethnicity = Asian, mother’s nativity = born inside the US, mothers education attainment = less than a college degree, WIC status = never a WIC recipient). Cumulative food insecurity is the number of times someone was food insecure, with a higher score indicating they were food insecure more. 202 youth experienced food insecurity 0 times, 45 experienced food insecurity once, 30 experienced food insecurity twice, 20 experienced food insecurity three times, and 24 experienced food insecurity four times. Coefficients are unstandardized.

Table 2 and Figure 2 present the results of the unadjusted and adjusted linear regressions assessing the association between cumulative food insecurity and youth food approach and food avoidance scores. For both outcomes, all model assumptions were met in the unadjusted and adjusted analysis. In the unadjusted analysis, a one-point increase in food insecurity, indicative of experiencing food insecurity more, was associated with an increase in youth’s food approach score (B = 0.32, 95% CI [0.11, 0.53], *p* = 0.003, R^2^ = 0.03) but was not significantly associated with youth’s food avoidance score (B = 0.04, 95% CI [−0.10, 0.18], *p* = 0.61, R^2^ = 0.001). Results were similar in the adjusted analysis for both food approach score (B = 0.30, 95% CI [0.04, 0.57], *p* = 0.03, R^2^ = 0.14) and food avoidance score (B = −0.03, 95% CI [−0.21, 0.15], *p* = 0.74, R^2^ = 0.10).

### 3.2. Interaction Between Food Insecurity and Lactation Intensity Ratio on Food Approach

There was a significant interaction between cumulative food insecurity and lactation intensity ratio in predicting food approach score (interaction: B = −0.06, 95% CI [−0.10, −0.01], *p* = 0.02, R^2^ = 0.16), but not between cumulative food insecurity and lactation intensity ratio in predicting food avoidance score (B = 0.02, 95% CI [−0.01, 0.05], *p* = 0.27, R^2^ = 0.11). For both outcomes, all model assumptions were met.

### 3.3. Interaction Between Food Insecurity and Clinically Relevant Breastfeeding Groups on Food Approach

There was a significant interaction between cumulative food insecurity and breastfeeding duration on food approach scores (B = −0.74, 95% CI [−1.18, −0.30], *p* = 0.001, R^2^ = 0.13). The interaction between cumulative food insecurity and breastfeeding intensity was not significant (B = 0.29, 95% CI [−0.23, 0.82], *p* = 0.27, R^2^ = 0.09). Given that the interaction between cumulative food insecurity and lactation intensity ratio on food avoidance was not significant, no further analysis on the clinically relevant breastfeeding groups was conducted.

### 3.4. Food Insecurity and Food Approach, Stratified by Breastfeeding Duration

**Figure 3:**
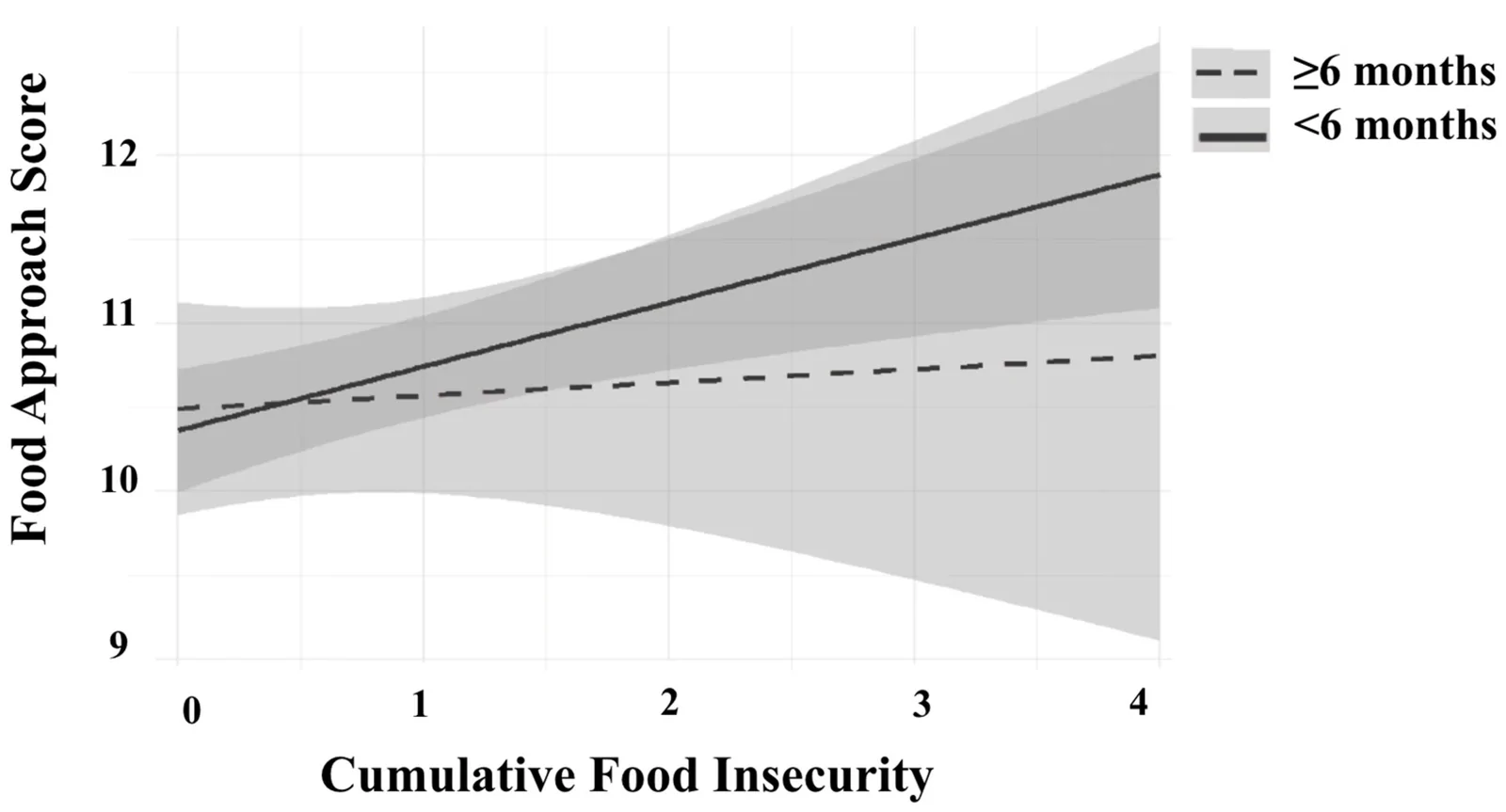
Association Between Cumulative Food Insecurity and Food Approach Score by Breastfeeding Duration. Cumulative food insecurity is the number of times someone was food insecure, with a higher score indicating they were food insecure more. Breastfeeding duration is categorized into those who were breastfed for <6 months (*n* = 109) versus those who were breastfed for ≥6 months (*n* = 212). The shaded portion represents the 95% confidence interval. Models adjusted for child’s BMI percentile, race and ethnicity, age, sex, mothers’ pre-pregnancy BMI, mother’s education attainment, WIC status, and mother’s nativity.

Table 3 and Figure 3 display the unadjusted and adjusted association between cumulative food insecurity and food approach score, stratified by breastfeeding duration. All model assumptions were met for each exploratory analysis. When stratified by breastfeeding duration, among youth who were breastfed for <six months, cumulative food insecurity was positively associated with the youth’s food approach score in the unadjusted (B = 0.69, 95% CI [0.37, 1.01], *p* < 0.001, R^2^ = 0.14) and adjusted (B = 0.73, 95% CI [0.37, 1.09], *p* < 0.001, R^2^ = 0.32) models. However, in both the unadjusted and adjusted models, among youth who were breastfed for ≥six months, cumulative food insecurity was not significantly associated with the youth’s food approach score in either the unadjusted (B = −0.03, 95% CI [−0.32, 0.26], *p* = 0.82, R^2^ = 0.000) or adjusted (B = −0.26, 95% CI [−0.65, 0.13], *p* = 0.18, R^2^ = 0.12).

### 3.5. Current Food Insecurity and Food Approach

In the unadjusted analysis, current food insecurity status was not significantly associated with food approach scores (B = 0.43, 95% CI [−0.20, 1.06], *p* = 0.18, R^2^ = 0.002). This relationship remained after adjusting for child’s BMI percentile, race and ethnicity, age, sex, mothers’ pre-pregnancy BMI, lactation intensity ratio, mother’s education attainment, WIC status, and mother’s nativity (B = 0.31, 95% CI [−0.44, 1.07], *p* = 0.41, R^2^ = 0.09).

## 4. Discussion

This study found that among youth previously exposed to gestational diabetes in utero, being exposed to food insecurity more often from birth to 11–16 years old was associated with higher food approach scores at 11–16 years old. However, once stratified by breastfeeding duration, this relationship was no longer significant among those who were breastfed for at least six months. Further, sensitivity analysis found that the relationship between current food insecurity status and food approach scores was not significant. These findings suggest that while repeated exposure to food insecurity across childhood is linked to adverse eating patterns, breastfeeding for at least six months may alter this relationship.

This study found that cumulative food insecurity was positively related to food approach, which is associated with a higher BMI and risk of developing binge-eating. This aligns with prior studies that have reported food insecurity to be associated with adverse eating behaviors. A longitudinal study of over 10,000 adolescents found that experiencing food insecurity was associated with a higher risk for binge-eating and otherwise-specified feeding and eating disorders two years later [38]. Similarly, a cross-sectional study of 194 children aged 8–10 years old found that child-reported food insecurity was associated with food preoccupation and more social pressure to eat, as parents in food-insecure households are more likely to use pressured feeding practices [39]. However, neither study controlled for the child’s BMI [38,39], which is a limitation, as eating behaviors are strongly related to BMI [40].

The development of the eating behaviors associated with a high food approach score may stem from a pattern of low food availability, followed by periods of high food availability, known as the feast-or-famine cycle, which is common among individuals with food insecurity [41,42]. This is especially true among individuals enrolled in the Supplemental Nutrition Assistance Program who have ample funds at the beginning of the month to purchase the quantity and quality of food they want [41,42]. However, by the end of the month, when their funds are depleted, they reduce their intake or purchase inexpensive foods to stretch their dollar [41,42]. Living in the feast-or-famine cycle has been associated with adverse eating behaviors, such as overeating when food is available [41]. This pattern of eating is problematic, as the inexpensive foods that are available in today’s food environment are ultra-processed foods that are high in calories, refined grains, added sugars, and low in fiber, protein, and whole grains [43,44]. The combination of an eating pattern adapted to overeating with the highly available energy-dense, inexpensive foods may lead to heightened food approach behaviors, which may be one mechanism between food insecurity and a higher risk for adverse health outcomes.

This study also found that the relationship between cumulative food insecurity and higher food approach scores was only among those who were breastfed for less than the recommended time (<six months). This is a novel and important finding, as it suggests that early life feeding may alter the relationship between food insecurity and adverse eating behaviors. Breastmilk has hormones, such as leptin, that are not found in formula, and have been associated with a lower risk for overweight and obesity later in life [45,46]. In addition, previous research has found that children who were breastfed for longer are more likely to consume fruits and vegetables and less likely to consume sugar-sweetened beverages, which is a dietary pattern associated with a reduced risk for overweight and obesity [35,47,48]. Longer breastfeeding durations are also associated with lower food approach scores and a higher use of responsive feeding practices [49,50,51]. Responsive feeding practices have been shown to instill healthy eating behaviors in children, such as regulating the amount they consume [52], which may make them more resistant to developing the feast-or-famine eating behavior associated with food insecurity. Mothers with higher educational attainment and who are healthier are more likely to exclusively breastfeed for longer and practice responsive feeding practices, suggesting some social factors may influence the relationship between breastfeeding and the risk for overweight and obesity [53,54]. Therefore, when it is feasible for the mother and offspring, encouraging mothers to breastfeed for at least six months may alter the association between cumulative food insecurity and adverse eating patterns.

These findings highlight the importance of consistently screening for food insecurity, as it provides a more complete picture of one’s experience with food insecurity. Currently, screening for food insecurity is not mandated in the US [55]. However, given that food insecurity is a social determinant of health, organizations such as the American Academy of Pediatrics have recommended screening for food insecurity during clinic visits [56]. Clinics that have implemented food insecurity screening have found it to be successful, as the screening was completed in almost every visit (98%) [57]. In addition, a study found that 75% of the patients who screened positively for food insecurity were later connected with a hunger relief organization [58]. Therefore, implementing regular screening may help connect those in need with resources that will help reduce food insecurity and its health consequences.

### Strengths and Limitations

This study benefited from the multiple measures of food insecurity. The study also accounts for many factors that may influence a child’s eating behavior, such as BMI and early life feeding. Further, the long-term prospective design of the racially and ethnically diverse cohort provides insight into how breastfeeding impacts eating behaviors later in life. In addition, this study was conducted in a cohort of offspring exposed to gestational diabetes in utero. This adds to the clinical significance of the findings, as these youth have a higher risk for developing poor cardiometabolic health. Finally, this study contributes to the limited understanding of the mechanisms linking food insecurity to adverse health outcomes by examining its relationship with adverse eating behaviors.

However, the study is not without limitations. First, since the sample was all youth exposed to gestational diabetes in utero, the generalizability of the relationship between cumulative food insecurity and eating behaviors among those not exposed to gestational diabetes in utero is limited. Second, while we controlled for variables relevant to food insecurity and this population, there may be other confounding variables that were not included. Third, eating behavior and food insecurity were reported by the mothers of the youth. While this is in line with the measures, self-report and shared-method bias could have influenced the results. Further, those in the analytic sample had a significantly higher lactation intensity ratio score, and a larger proportion were breastfed for six months or longer than those in SWIFT who were not included in the analytic sample. In addition, compared to those not included in the analytic sample, those in the analytic sample had a significantly higher prevalence of mothers with a college degree or more. Research has shown that mothers with a high educational attainment are more likely to exclusively breastfeed [59], meaning the analytic sample could have been biased. Therefore, the interaction between food insecurity and breastfeeding behavior could be inflated or distorted, and should be interpreted as an exploratory, hypothesis-generating finding. Finally, although food insecurity was measured four times, there was time in between the measurements in which experiences of food insecurity could have occurred, but was not captured by the survey.

## 5. Conclusions

Among youth exposed to gestational diabetes in utero, cumulative food insecurity during childhood was related to a high food approach score at 11–16 years old, which has been associated with a higher BMI. In exploratory analysis, breastfeeding for at least six months attenuated the relationship between food insecurity and food approach scores. This study is one of the few studies to analyze how cumulative food insecurity relates to eating behaviors, which has been a proposed mechanism behind the poor health outcomes associated with food insecurity. Future research is needed to understand the relationship between cumulative food insecurity and eating behaviors among youth not exposed to gestational diabetes in utero, and how the specific timing of when food insecurity occurs may influence eating behaviors.

## Figures and Tables

**Table 1:** Sociodemographic characteristics of the SWIFT baseline sample and differences between those not included in the analytic sample and those in the analytic sample.

	SWIFT Baseline Sample	Not in the Analytic Sample	Analytic Sample	*p*-Value
	*n* = 1033	*n* = 712	*n* = 321	
		N (%)		
Pre-pregnancy BMI (kg/m^2^)	29.65 (7.21)	29.88 (7.30)	29.16 (7.00)	0.13
GDM diagnosis (week)	25.32 (7.16)	25.36 (7.31)	25.23 (6.82)	0.78
GDM severity (z-scores sum)	0.10 (2.85)	0.20 (3.00)	−0.11 (2.48)	0.08
Total gestational weight gain				0.72
Less than recommended	327 (31.66%)	228 (32.02%)	99 (30.84%)	
Within recommendations	358 (34.66%)	241 (33.85%)	117 (36.45%)	
More than recommended	348 (33.69%)	243 (34.13%)	105 (32.71%)	
WIC				0.84
Never a WIC recipient	714 (69.12%)	494 (69.38%)	220 (68.54%)	
WIC recipient at some point	319 (30.88%)	218 (30.62%)	101 (31.46%)	
Education attainment				**0.002**
Less than college degree	529 (51.21%)	388 (54.49%)	141 (43.93%)	
College degree or more	504 (48.79%)	324 (45.51%)	180 (56.07%)	
Nativity				0.16
Outside the US/unknown	521 (50.44%)	370 (51.97%)	151 (47.04%)	
Inside the US	512 (49.56%)	342 (48.03%)	170 (52.96%)	
Insurance				0.27
Self/Employer	967 (93.61%)	662 (92.98%)	305 (95.02%)	
MediCal/Medicaid/Military	66 (6.39%)	50 (7.02%)	16 (4.98%)	
Youth’s Characteristics				
Sex				0.35
Male	549 (53.15%)	371 (52.11%)	178 (55.45%)	
Female	484 (46.85%)	341 (47.89%)	143 (44.55%)	
Race and Ethnicity				0.59
White	208 (20.14%)	139 (19.52%)	69 (21.50%)	
Asian	357 (34.56%)	243 (34.13%)	114 (35.51%)	
Hispanic	360 (34.85%)	250 (35.11%)	110 (34.27%)	
Black/Multiracial/Other	108 (10.45%)	80 (11.24%)	28 (8.72%)	
Size for Gestational Age				0.06
Average	784 (75.90%)	532 (74.72%)	252 (78.50%)	
Large	159 (15.39%)	122 (17.13%)	37 (11.53%)	
Small	90 (8.71%)	58 (8.15%)	32 (9.97%)	
Breastfeeding exclusivity				0.09
Exclusive	219 (21.20%)	140 (19.66%)	79 (24.61%)	
Not exclusive	814 (78.80%)	572 (80.34%)	242 (75.39%)	
Breastfeeding duration ^1^				**<0.001**
<six months	438 (42.48%)	329 (46.34%)	109 (33.96%)	
≥six months	593 (57.52%)	381 (53.66%)	212 (66.04%)	
	Mean (SD)			
Youth adolescence age (years) ^2^	13.53 (1.04)	13.55 (1.02)	13.52 (1.04)	0.83
Lactation intensity ratio (12 month)	5.73 (4.49)	5.27 (4.39)	6.76 (4.55)	**<0.001**

Note: To assess significant differences between SWIFT participants not included in the analytic sample and those included in the analytic sample, *t*-tests were used for continuous variables, Wilcoxon rank sum for non-normally distributed continuous variables, and chi-square for categorical variables. Bolded values indicate significance at *p* < 0.05. ^1^ *n* = 1031. ^2^ *n* = 414. GDM is gestational diabetes. WIC is the Special Supplemental Nutrition Program for Women, Infants, and Children.

**Table 2:** Unadjusted and adjusted linear regression assessing how cumulative food insecurity is associated with children’s food approach and food avoidance score.

	Unadjusted			Adjusted		
	B	95% CI	*p*	B	95% CI	*p*
Food approach						
Cumulative food insecurity	0.32	[0.11, 0.53]	**0.003**	0.30	[0.04, 0.57]	**0.03**
Food avoidance						
Cumulative Food insecurity	0.04	[−0.10, 0.18]	0.61	−0.03	[−0.21, 0.15]	0.74

*n* = 321. Cumulative food insecurity is the number of times someone was food insecure, with a higher score indicating they were food insecure more. Models adjusted for child’s BMI percentile, race and ethnicity, age, sex, lactation intensity ratio, mother’s pre-pregnancy BMI, mother’s education attainment, WIC status, and mother’s nativity. Bolded values indicate significance at *p* < 0.05. Unstandardized coefficients are presented.

**Table 3:** Unadjusted and adjusted linear regression assessing how cumulative food insecurity is associated with children’s food approach score between breastfeeding duration groups.

Food Approach	Unadjusted			Adjusted		
	B	95% CI	*p*	B	95% CI	*p*
Breastfeeding Duration						
Breastfed <6 months (*n* = 109)	0.69	[0.37, 1.01]	**<0.001**	0.73	[0.37, 1.09]	**<0.001**
Breastfed ≥6 months (*n* = 212)	−0.03	[−0.32, 0.26]	0.82	−0.26	[−0.65, 0.13]	0.18

Cumulative food insecurity is the number of times someone was food insecure, with a higher score indicating they were food insecure at the times it was measured. Breastfeeding duration is categorized into those who were breastfed for <6 months (*n* = 109) versus those who were breastfed for ≥6 months (*n* = 212). Models adjusted for child’s BMI percentile, race and ethnicity, age, sex, mothers’ pre-pregnancy BMI, mother’s education attainment, WIC status, and mother’s nativity. Unstandardized coefficients are presented. Bold values indicate significance at *p* < 0.01.

## Data Availability

Data is available upon reasonable request from the corresponding author.
